# Transcriptome analysis reveals response regulator SO2426-mediated gene expression in *Shewanella oneidensis *MR-1 under chromate challenge

**DOI:** 10.1186/1471-2164-9-395

**Published:** 2008-08-21

**Authors:** Karuna Chourey, Wei Wei, Xiu-Feng Wan, Dorothea K Thompson

**Affiliations:** 1 Environmental Sciences Division, Oak Ridge National Laboratory, Bethel Valley Road, Oak Ridge, TN 37831, USA; 2 Department of Biological Sciences, Purdue University, 915 W. State Street, West Lafayette, IN 47907, USA; 3 Systems Biology Laboratory, Department of Microbiology, Miami University, 32 Pearson Hall, Oxford, OH 45056, USA; 4 Chemical Sciences Division, Oak Ridge National Laboratory, Oak Ridge, TN 37831, USA; 5 Influenza Division, Centers for Disease Control and Prevention, 1600 Clifton Road, Atlanta, GA 30333, USA

## Abstract

**Background:**

*Shewanella oneidensis *MR-1 exhibits diverse metal ion-reducing capabilities and thus is of potential utility as a bioremediation agent. Knowledge of the molecular components and regulatory mechanisms dictating cellular responses to heavy metal stress, however, remains incomplete. In a previous work, the *S. oneidensis so2426 *gene, annotated as a DNA-binding response regulator, was demonstrated to be specifically responsive at both the transcript and protein levels to acute chromate [Cr(VI)] challenge. To delineate the cellular function of SO2426 and its contribution to metal stress response, we integrated genetic and physiological approaches with a genome-wide screen for target gene candidates comprising the SO2426 regulon.

**Results:**

Inactivation of *so2426 *by an in-frame deletion resulted in enhanced chromate sensitivity and a reduced capacity to remove extracellular Cr(VI) relative to the parental strain. Time-resolved microarray analysis was used to compare transcriptomic profiles of wild-type and SO2426-deficient mutant *S. oneidensis *under conditions of chromate exposure. In total, 841 genes (18% of the arrayed genome) were up- or downregulated at least twofold in the Δ*so2426 *mutant for at least one of six time-point conditions. Hierarchical cluster analysis of temporal transcriptional profiles identified a distinct cluster (n = 46) comprised of co-ordinately regulated genes exhibiting significant downregulated expression (*p *< 0.05) over time. Thirteen of these genes encoded proteins associated with transport and binding functions, particularly those involved in Fe transport and homeostasis (*e.g*., siderophore biosynthetic enzymes, TonB-dependent receptors, and the iron-storage protein ferritin). A conserved hypothetical operon (*so1188*-*so1189*-*so1190*), previously identified as a potential target of Fur-mediated repression, as well as a putative bicyclomycin resistance gene (*so2280*) and cation efflux family protein gene (*so2045*) also were repressed in the *so2426 *deletion mutant. Furthermore, the temporal expression profiles of four regulatory genes including a *cpxR *homolog were perturbed in the chromate-challenged mutant.

**Conclusion:**

Our findings suggest a previously unrecognized functional role for the response regulator SO2426 in the activation of genes required for siderophore-mediated Fe acquisition, Fe storage, and other cation transport mechanisms. SO2426 regulatory function is involved at a fundamental molecular level in the linkage between Fe homeostasis and the cellular response to chromate-induced stress in *S. oneidensis*.

## Background

*Shewanella oneidensis *MR-1, a facultatively anaerobic member of the γ-class of *Proteobacteria*, can respire anaerobically by reducing a wide variety of alternative electron acceptors including fumarate, nitrate, elemental sulfur, and such oxidized metals as Fe(III), Mn(IV), and U(VI) [1-5]. In addition, MR-1 is capable of reducing hexavalent chromium, or chromate [Cr(VI)], to less toxic and sparingly soluble trivalent chromium [Cr(III)] under both aerobic and anaerobic conditions [6-8]; however, there have been no reports to date of the ability of MR-1 to generate energy for growth using Cr(VI) as the sole terminal electron acceptor for anaerobic respiration [9]. This plasticity in the use of alternative electron acceptors for anaerobic respiration has engendered interest in *S. oneidensis *MR-1 as a model environmental organism with potential utility in the bioremediation of dissolved metal ions, and as a consequence, the complete MR-1 genome was sequenced to advance understanding of *Shewanella *biology [10].

To expand knowledge of metal stress responses in particular, we previously initiated genome-based studies focused on identifying the molecular components involved in the *S. oneidensis *response to chromate [11-13], an anthropogenic pollutant widely distributed in the environment due to its prevalent use in manufacturing and military industries [14,15]. Chromate toxicity is associated with the generation of reactive oxygen species during the intracellular partial reduction of Cr(VI) to the highly reactive radical Cr(V) by various *in vivo *nonspecific reductants or cellular one-electron reductases [16]. Our previous work employing DNA microarrays and multidimensional liquid chromatography-tandem mass spectrometry (LC-MS/MS) demonstrated that a functionally unknown DNA-binding response regulator (designated SO2426) in MR-1 was reproducibly and significantly upregulated at both the transcript and protein levels in response to acute chromate challenge [11,13].

In accordance with its ability to adapt to various environmental conditions, the *S. oneidensis *MR-1 genome encodes a relatively large repertoire of transcriptional regulators, including 88 predicted two-component regulatory system proteins consisting of 23 histidine protein kinase (HK) genes, 57 response regulators (RR), and 8 HK-RR hybrids [10]. Prototypical two-component systems, which constitute the predominant mechanism used by bacteria for coupling environmental signals to specific adaptive responses, comprise a sensor histidine kinase and a cognate response regulator [17-19]. Fundamental to signal transduction is a phosphotransfer mechanism that forms the core of both mechanistically simple as well as more complex signaling pathways. In this scheme, a membrane-bound sensor kinase catalyzes ATP-dependent autophosphorylation at a conserved histidine residue in the HK cytoplasmic autokinase domain in response to a specific environmental cue. The signal is transduced through transfer of the phosphoryl group from the phosphoHis of the HK to a conserved aspartate residue within the receiver module of a cytosolic RR. Phosphorylation of the RR alters its affinity for DNA, leading to the transcriptional activation or repression of specific genes. An essential feature of two-component systems is that specific histidine kinases and response regulators function as cognate pairs and are often arranged within the same operon. The *S. oneidensis *SO2426 protein appears to be an orphan RR as defined by the fact that there is no obvious cognate histidine kinase flanking the *so2426 *gene or proximally located based on the genome annotation [10].

The primary aim of the present study was to investigate the function of the uncharacterized SO2426 regulator within the context of chromate stress by performing a genome-wide screen for potential target genes of this RR in *S. oneidensis *MR-1. Toward this end, we created an *so2426 *in-frame deletion mutant and compared its global transcriptional profiles with that of wild-type MR-1 in response to chromate challenge using time-resolved microarray experiments. Comparative transcriptome analysis suggested that SO2426 was associated with the activation of genes involved in siderophore-mediated Fe uptake, Fe storage, and possibly other cation transport processes.

## Results and discussion

### Sequence analysis of the response regulator SO2426

**Figure 1:**
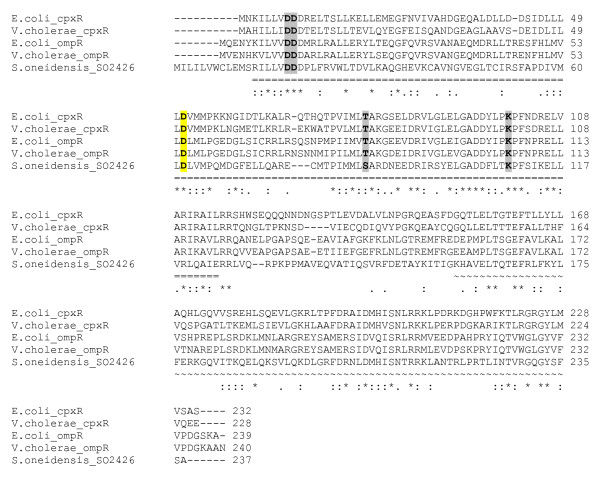
**Sequence alignment of *S. oneidensis *SO2426 with other two-component response regulators**. ClustalW [20] was used to perform a multiple sequence alignment consisting of *S. oneidensis *SO2426 (GenBank ID 24348419), CpxR from *E*. *coli *(GenBank ID 16131752) and *Vibrio cholerae *(GenBank ID 147675245), and OmpR from *E*. *coli *(GenBank ID 16131282) and *V*. *cholerae *(GenBank ID 147673571). The region underlined with "=" is the aligned regulator receiver domain with predicted domain (SO2426: positions 13–124), and the region denoted with "~" is the aligned C-terminal domain containing the wHTH DNA-binding motif (SO2426: positions 158–235). Boldface letters highlighted in grey indicate conserved signature residues of receiver domains [19]. Residue D62 is predicted as 4-aspartylphosphate, the putative phosphorylation site (highlighted in yellow). The star, colon, and dot notations rank the sequence conservation from high to low.

Based on its nucleotide sequence, the *so2426 *gene of *S. oneidensis *MR-1 is predicted to encode a 237-amino-acid protein with a calculated molecular mass of 27.4 kDa [10]. Comparative sequence analysis using ClustalW [20] revealed that SO2426 shares sequence similarity to CpxR from *Vibrio cholerae *(36% identity) and *E. coli *(34%), and to OmpR from *V*. *cholerae *(29%) and *E. coli *(27%) (Figure 1). In addition, SO2426 orthologs are present and conserved among many of the *Shewanella *species sequenced to date, including *Shewanella *sp. MR-7 (88% sequence identity), *Shewanella *sp. MR-4 and sp. ANA-3 (87%), *S. baltica *OS195 (74%), *S. putrefaciens *200 and CN-32 (73%), *S. woodyi *ATCC 51908 (60%), *S. amazonensis *SB2B (61%), and *S. frigidimarina *NCIMB 400 (49%).

The deduced SO2426 protein consists of an N-terminal CheY-like receiver domain and a C-terminal winged-helix DNA-binding domain. The N-terminal portion of SO2426 (amino acids 13–124; see Figure 1) shares homology with receiver domains and contains the highly conserved signature residues that are thought to constitute the phosphorylated acid-pocket active site [19,21]. In CheY, these residues correspond to Asp12, Asp13, and Asp57, with Thr87 and Lys109 completing the cluster of conserved residues surrounding the phosphorylation site [19]. Thr87 and Lys109, although not absolutely required for phosphorylation, have been implicated in contributing to the phosphorylation-induced conformational change [22-24]. *S. oneidensis *SO2426 contains three contiguous aspartate residues near its N-terminus at positions 18, 19 and 20, and there is a conserved substitution to serine at the equivalent position of 87. The phosphate-accepting aspartic residue Asp57 and Lys109 (relative to CheY residue numbering) are retained at equivalent positions in the SO2426 primary sequence. It is presently not known whether a cognate histidine kinase interacts with SO2426 or whether phosphorylation plays a regulatory role in the cellular activity of SO2426. The fact that SO2426 contains the highly conserved residues constituting the active phosphorylation pocket (D18, D19, D62, and K109) suggests that the protein might be differentially controlled by phosphorylation. Furthermore, it remains to be determined whether the regulatory activity of SO2426 is modulated in response to environmental or intracellular stimuli.

The C-terminal portion of SO2426 (amino acids 158–235; see Figure 1) contains a predicted winged helix-turn-helix (wHTH) motif indicative of the DNA-binding domains of response regulators in the OmpR/PhoB subfamily [25-27]. The basic structure of the carboxy-terminal domains of OmpR/PhoB subfamily response regulators is characterized by an amino-terminal four-stranded β sheet, a central three-helical bundle, and a C-terminal β-strand hairpin (the wing), which provides an additional interface for DNA contact [26].

### The *so2426 *gene is co-transcribed in an operon

**Figure 2:**
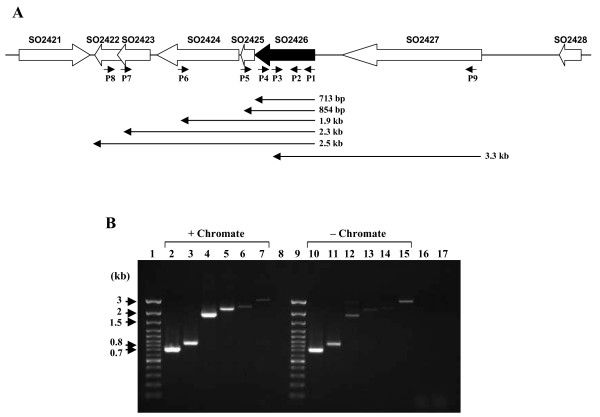
**Structural organization of the *S. oneidensis *MR-1 *so2426 *locus**. (A) Schematic representation of the *so2426 *gene region. ORFs located upstream and downstream of *so2426 *(black arrow) are indicated by open arrows, which also indicate the direction of transcription. The deduced proteins of the ORFs flanking *so2426 *have the following annotations based on the J. Craig Venter Institute's Comprehensive Microbial Resource : SO2421 is a L-asparaginase I; SO2422 is a conserved hypothetical protein; SO2423 is a hypothetical protein; SO2424 is a zinc carboxypeptidase domain protein; SO2425 is a hypothetical protein; SO2426 is a DNA-binding response regulator; SO2427 is a putative TonB-dependent receptor; and SO2428 is a hypothetical protein. The locations of the oligonucleotide primers (P1–P9) used for RT-PCR are shown by the small solid arrows. The expected PCR product sizes for the different primer pairs are given below. (B) Agarose gel (1%) electrophoresis of amplified DNA fragments derived from *S*. *oneidensis *MR-1 cDNA templates under conditions of chromate stress or no stress. Lane designations: (1) 100-bp DNA ladder; (2) PCR primer pair P1/P4, (3) P1/P5, (4) P1/P6, (5) P1/P7, (6) P2/P8, (7) P9/P3, (8) blank, (9) 100-bp DNA ladder, (10) P1/P4, (11) P1/P5, (12) P1/P6, (13) P1/P7, (14) P2/P8, (15) P9/P3, (16) chromate-treated total RNA used as the template in PCR amplification with P1/P4 (negative control), and (17) non-stressed total RNA used as the template in PCR amplification with P1/P4 (negative control).

Based on the MR-1 genome annotation, SO2426 is an apparent orphan response regulator given that the *so2426 *locus is not linked with a gene that encodes a potential cognate histidine kinase. As schematically represented in Figure 2A, *so2426 *is tightly clustered with four functionally unknown downstream genes, all possessing the same transcriptional polarity. Located immediately downstream of *so2426 *with a short intergenic spacer of 10 bp is a small ORF (132 bp) predicted to encode a soluble hypothetical protein (SO2425), which was shown previously to be induced at the transcriptional level in response to acute chromate challenge [11]. The gene cluster is also characterized by ORFs encoding a zinc carboxypeptidase domain protein (SO2424), a hypothetical protein (SO2423), and a conserved hypothetical protein (SO2422) (Figure 2A). The *so2423 *ORF overlaps *so2422 *by 3 bp based on the MR-1 genome annotation (). The *so2426 *region also includes a gene encoding a putative TonB-dependent receptor (SO2427) positioned approximately 170 bp upstream of *so2426*. Putative ρ-independent transcription terminators were identified downstream of the *so2427 *and *so2422 *ORFs (; [28]).

Reverse transcription-PCR (RT-PCR) experiments were performed to determine whether the *so2427*-*so2422 *gene cluster is transcribed as a polycistronic mRNA. Primers P1–P9 (Figure 2A) used in the PCR reactions following reverse transcription with random hexamers were designed to amplify regions spanning two or more ORFs in order to establish the transcriptional organization. To assess the reproducibility of the results, multiple independent RT-PCR experiments were carried out using different preparations of total RNA isolated from untreated or chromate-treated MR-1 cells. Figure 2B shows a representative gel of the RT-PCR results using the entire suite of primers. Each PCR product was confirmed by DNA sequencing. No products were amplified when using either total RNA as the template for PCR amplification (Figure 2B) or primers complementary to an intergenic region located outside of the *so2427*-*so2422 *cluster (results not shown), thus indicating the absence of contaminating genomic DNA in the total RNA preparations. *S. oneidensis *MR-1 genomic DNA was used as a positive control for the PCR conditions (results not shown). Amplicons of the expected sizes, *i*.*e*., 0.85 kb for primers P1/P5, 1.9 kb for primers P1/P6, 2.3 kb for primers P1/P7, 2.5 kb for primers P2/P8, and 3.3 kb for primers P9/P3, were obtained (Figure 2B), indicating that the *so2427*-*so2422 *region is co-transcribed as a polycistronic mRNA under no-metal and chromate-amended conditions. However, given the presence of a putative ρ-independent terminator immediately downstream of *so2427*, we cannot rule out the possibility that the product generated from the *so2427*-*so2426 *transcript might be due to read-through transcription.

### Characterization of the *so2426 *promoter region

**Figure 3:**
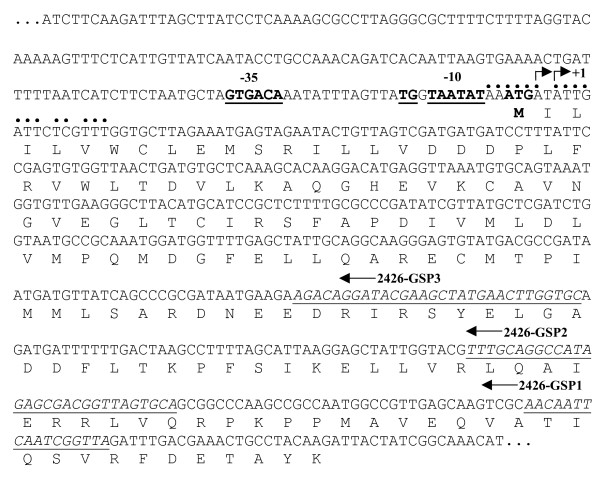
**Nucleotide sequence of the *so2426 *promoter region and N-terminal coding region**. The transcriptional start site (+1) is indicated by arrows, and putative -10 and -35 basal promoter elements are presented in boldface and underlined. Closed circles indicate conserved residues matching a predicted Fur-binding recognition site. The annotated (original) translation start codon (M1 residue) of *so2426 *is shown in boldface. 5'-RACE results described here indicate that the annotated translation start codon is incorrect, and residue M11 constitutes the actual start codon. The 5'-RACE primers used to identify the transcription start site are shown in italics and underlined.

The transcription start site of the *so2426 *gene was localized using 5' RACE analysis of mRNA transcripts isolated from wild-type cells grown in either the absence or presence of chromate. Two clear 5' termini, both mapping to A residues and corresponding to positions 25 and 27 bp upstream of a second in-frame ATG (amino acid residue M11) (Figure 3), were obtained by DNA sequencing of the 5' RACE amplicons. These sites are located at positions 4 and 6 bp downstream of the initial ATG (M1) start codon, indicating that the original sequence annotation for this gene is incorrect and that the second ATG (M11) most likely constitutes the translation start codon for SO2426. The *so2426 *transcription start point indicated potential -10 and -35 basal promoter elements, 5'-TAatAT and 5'-gTGACA, respectively, with a 15-bp spacing between them. The putative -10 promoter sequence (5'-TAatAT) has a 4/6 match to the consensus σ^70^-dependent -10 hexamer element (5'-TATAAT) from *E. coli*. Interestingly, located one base upstream of the -10 hexamer is the extended -10 element 5'-TaTG-3' (Figure 3), identified by Mitchell *et al*. [29] and shown to be an important determinant for promoter activity. Finally, the identified *so2426 *promoter region contains the putative -35 element 5'-gTGACA, which is a strong match (5/6) to the *E. coli *consensus -35 hexamer (5'-TTGACA).

Further analysis of the *so2426 *upstream regulatory region revealed the presence of a putative recognition site for the ferric uptake regulator (Fur), the iron-binding global transcriptional regulator that serves as the dominant sensor of Fe availability in both gram-positive and gram-negative bacteria (for a review, see [30]). A putative palindromic Fur box, 5'-AAATGAtATTgATTcTCgTTT-3', which closely matched our previously derived predicted Fur-binding motif for *S. oneidensis *MR-1 [31], was identified at positions -5 to +16 immediately downstream of the -10 promoter element and overlapped the mapped transcription start sites for *so2426 *(Figure 3). Identification of a Fur box is consistent with our previous microarray studies demonstrating increased *so2426 *mRNA levels in a *S. oneidensis *MR-1 *fur *knockout strain [31] and suggests that expression of the SO2426 response regulator might be subject to Fur-mediated repression.

### Growth and chromate reduction kinetics of the *so2426 *deletion mutant

**Figure 4:**
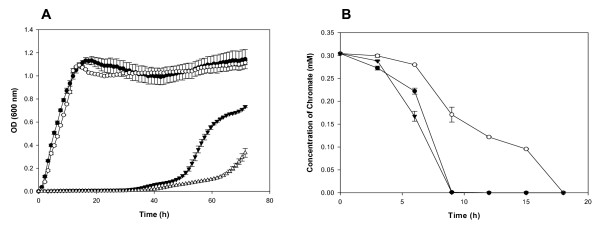
**Growth and chromate reduction kinetics of *S. oneidensis *MR-1 wild type and the Δ*so2426 *mutant**. (A) Wild-type (●) and mutant (○) cultures were cultivated in LB broth without the addition of chromate under aerobic conditions and compared to wild-type (▼) and mutant (△) cultures grown in LB broth amended with a final K_2_CrO_4 _concentration of 0.3 mM. (B) Cr(VI) removal rates for the *S*. *oneidensis *MR-1 (●), Δ*so2426 *(○), and complemented Δ*so2426*(*so2426*^+^) (▼) strains in LB broth supplemented with 0.3 mM chromate. The different cultures were challenged with 0.3 mM K_2_CrO_4 _when cells reached an OD_600 _of 0.5 (mid-log point). The error bars indicate standard deviations of triplicate measurements.

A *cre*-*lox *recombination method [32,33] was used to create a nonpolar in-frame deletion of the *so2426 *locus in MR-1 for the purpose of analyzing in depth the functional role of this response regulator in metal stress responses (see Methods for a detailed description). The deleted segment was 721 bp in length and corresponded to positions -77 to +645 (*S. oneidensis *MR-1 genome coordinates 2534690 and 2533969) from the annotated translational start site, leaving only 69 nucleotides of the *so2426 *gene intact at the C-terminal end. The growth behavior of the Δ*so2426 *mutant in response to Cr(VI) was examined using a Bioscreen C reader to monitor culture turbidity (OD_600_) at 30-min intervals under aerobic conditions over 72 h. As shown in Figure 4A, growth of the *S. oneidensis *Δ*so2426 *strain was comparable to the wild type in LB broth without added chromate. By contrast, growth of the Δ*so2426 *mutant was inhibited to a greater extent than the wild type in the presence of 0.3 mM chromate as evident from the longer time interval required before the commencement of exponential growth.

To determine whether deletion of *so2426 *affected the ability of MR-1 to remove extracellular Cr(VI) from the medium, the wild-type and mutant strains were challenged with 0.3 mM chromate (the same final concentration used for the transcriptomic studies) when cells reached mid-log phase (OD_600_, 0.5). Residual hexavalent chromium was measured spectrophotometrically at 3-h intervals using the chromogenic 1,5-diphenylcarbazide (DPC) method [34]. The chromate disappearance profiles in Figure 4B show that the parental MR-1 strain removed 100% of the external Cr(VI) within 9 h, while the Δ*so2426 *mutant transformed approximately 50% within the same time period. No abiotic conversion of chromate was observed for the cell-free LB control (data not shown).

Complementation experiments were performed to ascertain whether the enhanced chromate sensitivity and slower chromate transformation rate for the *so2426 *null mutant were due to the deletion of the response regulator gene *so2426 *and not to secondary-site mutations introduced during the mutagenesis procedure. A complemented Δ*so2426 *strain was constructed by cloning the full-length *so2426 *wild-type ORF plus 100 bp of contiguous upstream sequence into the broad-host-range vector pBBR2426 as described in the Methods. Transformation of pBBR2426 into the Δ*so2426 *mutant resulted in a complemented strain, designated Δ*so2426*(*so2426*^+^). Genetic complementation of the *so2426 *gene in the mutant strain restored growth (data not shown) and Cr(VI) reduction capacity to near wild-type levels (Figure 4B), thus reaffirming the integral role of SO2426 in the cellular response to chromate.

### Temporal transcriptome analysis of Δ*so2426 *and wild-type strains

Prior to this study, nothing was known about the regulatory targets of the OmpR family response regulator SO2426. To investigate the effect of the *so2426 *deletion on global gene expression and to identify candidate target genes under SO2426 control, the chromate-perturbed transcriptomes of the Δ*so2426 *mutant and the *S. oneidensis *MR-1 wild type were compared using whole-genome microarray analysis. Time-series microarray experiments were performed in which global transcriptional profiles of the wild type and the Δ*so2426 *mutant strains were compared at 5, 30, 60, 90, 180 min, and 24 hours following the addition of K_2_CrO_4 _(0.3 mM final concentration) to mid-exponentially growing cells (see Additional file 1 for a complete list of the processed microarray data). Genes with altered expression ratios of ≥ 2-fold for at least one of the time points and having a *p *value of < 0.05 were considered to be significantly changed and were further analyzed using clustering methods. Overall, microarray expression profiling revealed that 82 (5 min), 90 (30 min), 80 (60 min), 109, (90 min), 443 (180 min), and 465 (24 h) genes were induced in the Δ*so2426 *mutant under chromate exposure, whereas 125 (5 min), 81 (30 min), 56 (60 min), 105 (90 min), 314 (180 min), and 364 (24 h) genes were repressed. The total number of differentially expressed genes was substantially less at the shorter, acute exposure times (*i.e*., 5, 30, 60, and 90 min) compared to the longer exposure periods (*i.e*., 180 min and 24 h), at which point other effects such as accumulation of intracellular chromium (hexavalent and/or reduced forms) and stationary phase growth are likely impacting global expression profiles.

Quantitative real-time RT-PCR (qRT-PCR) analysis was employed to assess the general quality of the microarray data by providing an independent measurement of gene expression for a small subset of genes. The five genes (*so0404*, *so2426*, *so3670*, *so1826*, and *so3585*) selected for comparative qRT-PCR displayed downregulated or unaltered expression patterns at the 180-min time point of chromate exposure based on microarray hybridization. Comparison of gene expression levels as determined by microarray and qRT-PCR analyses indicated a high degree of concordance, with a Pearson's correlation coefficient (*r*) value of 0.99 (data not shown). For the mutant strain, both microarray hybridization and qRT-PCR indicated that *so2426 *expression levels were not detectable above background, thus providing additional confirmation of deletion of the *so2426 *gene.

### Functional classification of genes differentially expressed in the Δ*so2426 *mutant

**Figure 5:**
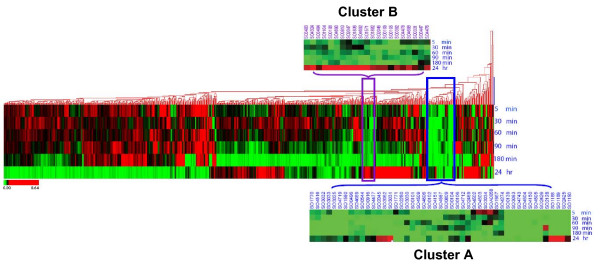
**Complete linkage clustering analysis of genes with altered expression profiles in the Δ*so2426 *mutant**. The 841 genes (out of the 4,648 represented on the microarray) exhibiting at least a twofold statistically significant (*p *< 0.05) change in expression for at least one of the time points during Cr(VI) exposure were analyzed by pairwise complete-linkage clustering. Transcriptional profiles are shown at 5, 30, 60, 90, 180 min, and 24 h post chromate addition. Individual genes are represented by a single row, and each exposure time interval is represented by a single column. Red represents induction, while green represents repression. Two noteworthy clusters (A and B) are indicated, with their respective expression heat maps enlarged.

Pairwise complete-linkage clustering was used to identify groups of potentially co-ordinately regulated genes among the 841 ORFs (18% of the arrayed genome) showing a twofold or greater statistically significant (*p *< 0.05) change in expression for at least one time point during Cr(VI) exposure. One particularly noteworthy cluster of similarly expressed genes (Cluster A in Figure 5) comprises 46 genes with functions distributed across the following role categories: cellular processes (2 genes), energy metabolism (9 genes), hypothetical proteins (17 genes), protein synthesis (1 gene), regulatory functions/signal transduction (4 genes), and transport and binding proteins (13 genes). These clustered genes displayed a distinct pattern of downregulated expression in the Δ*so2426 *mutant relative to the wild-type MR-1 strain, particularly at the earlier time points (5, 30, 60, and 90 min) of chromate exposure (see Table 1). This is consistent with our previous studies demonstrating that the wild-type *so2426 *gene was induced at the transcriptional level early in Cr(VI) exposure, *i.e*. 90 min or less, but not at 24 h post-treatment [11,12]. Closer inspection of the 46 potentially co-regulated genes (Table 1) revealed that many of the transport/binding, hypothetical, and regulatory genes were shown previously to be upregulated in wild-type MR-1 cells in response to chromate treatment [11,13], thus suggesting that these ORFs are candidate gene targets of positive control by SO2426. The genes constituting Cluster A are discussed in greater detail in the sections below.

**Table 1:** Selected genes with down-regulated expression profiles in the Δ*so2426 *mutant relative to wild-type MR-1

		**Mean (Δ*so2426*/WT) mRNA ratio^†^**					
		
		**Time (min) post Cr(VI) addition**					
		
**Gene**	**Description**	**5**	**30**	**60**	**90**	**180**	**1440**
**Cellular processes**							

SO2280	Bicyclomycin resistance protein	0.64	0.36	0.43	0.23	0.24	0.39
SO4274	Undecaprenol kinase, putative	0.67	0.7*	0.57	0.56*	0.8*	0.36

**Energy metabolism**							

SO0101	Formate dehydrogenase (*fdnG*)	0.3	0.32	0.55	0.56	0.29	0.36
SO0102	Formate dehydrogenase (*fdnH*)	0.37	0.31	0.72	0.53	0.25	0.67*
SO0104	FdhE protein (*fdhE*)	0.32	0.74*	0.45	0.61*	0.43	0.76*
SO0809	Azurin precursor (*azu*)	0.44	0.69	0.28	0.84	0.3	0.4
SO3034	Ferric iron reductase protein, putative	1.09*	0.61	0.91*	0.25	0.45	0.42
SO4151	Polysaccharide deacetylase family protein	0.29	0.3	0.35	0.6	0.24	0.23
SO4503	Formate dehydrogenase accessory protein FdhD	1.13*	0.56	0.53	0.5	0.28	0.36
SO4506	Iron-sulfur cluster-binding protein	0.42	0.28	0.77	0.45	0.47	0.39
SO4509	Formate dehydrogenase, alpha subunit	0.4	0.29	0.63	0.38	0.23	0.37

**Hypothetical**							

SOA0058	Hypothetical protein	1.19	0.82*	1.0*	0.37*	0.29	0.27
SO0496	Conserved hypothetical protein	0.59	0.4	0.48	0.45	0.46	0.66*
SO1188	Conserved hypothetical protein	0.01	0.013	0.007	0.006	0.004	1.52*
SO1189	Conserved hypothetical protein	0.003	0.022	0.006	0.004	0.005	1.68*
SO1190	Conserved hypothetical protein	0.01	0.014	0.02	0.009	0.02	1.03*
SO1770	Glycerate kinase, putative	0.56	0.48	0.35	0.18	0.22	0.72*
SO1967	Hypothetical protein	0.97*	0.78*	0.34	0.55*	0.72	0.42
SO2128	Hypothetical protein	0.38	0.43	0.32	1.21	0.54	0.89*
SO2425	Hypothetical protein	0.21	0.29	0.23	0.09	0.14	1.63*
SO2469	Conserved hypothetical protein	0.35	0.53	0.68	0.62	0.46	0.96*
SO3025	Conserved hypothetical protein	0.17	0.16	0.16	0.2	0.23	0.73*
SO3062	Hypothetical protein	0.48	0.15	0.16	0.14	0.21	1.21*
SO4502	Conserved domain protein	0.93*	0.74*	0.54	0.54*	0.26	0.3
SO4504	Conserved hypothetical protein	0.2	0.06	0.15	0.14	0.23	0.13
SO4505	Conserved hypothetical protein	0.23	0.16	0.45	0.23	0.29	0.16
SO4689	Conserved hypothetical protein	0.51	0.43	0.4	0.45	0.54	0.76*
SO4719	Conserved hypothetical protein	0.23	0.36	0.36	0.31	0.18	0.62*

**Protein synthesis**							

SO0106	Selenocysteine-specific translation elongation factor (*selB*)	0.38	0.72*	0.57	0.76*	0.46	0.83*

**Regulatory functions**							

SO0916	Transcriptional regulator, MarR family	0.64	0.42	0.4	0.34	0.33	0.84*
SO0544	Sensory box histidine kinase	0.53	0.31	0.35	0.51	0.52	0.58*
SO4477	Transcriptional regulatory protein CpxR (*cpxR*)	0.54	0.32	0.37	0.35	0.43	0.89*
SO4567	Transcriptional regulator, AsnC family	0.41	0.39	0.44	0.79	0.34	0.45

**Transport and binding proteins**							

SO0139	Ferritin (*ftn*)	0.2	0.22	0.11	0.08	0.05	0.19
SO1580	TonB-dependent heme receptor	0.32	0.39	0.37	0.19	0.23	0.92*
SO1771	Permease, GntP family	0.88*	0.23	0.43	0.37	0.14	0.2
SO2045	Cation efflux family protein	0.57	0.24	0.3	0.22	0.13	1.19*
SO3030	Siderophore biosynthesis protein AlcA (*alcA*)	0.67	0.27	0.81*	0.21	0.15	0.69*
SO3031	Siderophore biosynthesis protein, putative	0.52	0.34	0.3	0.16	0.08	1.37*
SO3032	Siderophore biosynthesis protein, putative	0.5	0.33	0.25	0.14	0.06	0.73*
SO3033	Ferric alcaligin siderophore receptor	0.73	0.34	0.29	0.17	0.07	0.77*
SO3063	Sodium:alanine symporter family protein	0.26	0.18	0.12	0.15	0.18	0.34
SO4150	Transporter, putative	0.07	0.12	0.11	0.34	0.27	0.3
SO4516	Ferric vibriobactin receptor (*viuA*)	0.59	0.54	0.37	0.21	0.16	0.67*
SO4712	ABC transporter, ATP-binding protein, putative	0.49	0.84*	0.59	0.77*	0.54	0.59*
SO4743	TonB-dependent receptor, putative	0.08	0.16	0.09	0.06	0.05	0.43

^†^The mRNA ratios (Δ*so2426 *mutant/wild type) per time interval represent mean values derived from six DNA microarray experiments performed with total RNA isolated from three independent cultures (plus two dye reversal reactions per biological replicate) in chromate-amended LB medium.

*Expression ratio values determined to be statistically non-significant (*p *> 0.05).

Cluster B (Figure 5) includes 20 genes, three of which encode AcrB/AcrD/AcrF family proteins (SO1882, SO3484, SO4692) presumably involved in drug efflux and resistance. All of these 20 genes showed decreased expression in the Δ*so2426 *mutant except at the 24-h time point, where mRNA levels increased (Figure 5 and Additional file 1). Notably, Cluster B also contains genes *so0392 *(putative lipoprotein) and *so4688 *(glycosyl transferase, group 2 family protein), both with annotated functions associated with the cell envelope, as well as *cpxA *(*so4478*). In *E. coli*, CpxA is the sensory inner membrane kinase that functions with its cognate partner, a cytoplasmic response regulator (CpxR), in a prototypical two-component system to sense and respond to perturbations in the bacterial cell envelope (reviewed in [35]). *S. oneidensis cpxR *also displayed downregulated temporal expression profiles in the Δ*so2426 *mutant under chromate challenge but grouped with genes in Cluster A.

### Genes encoding transport and binding functions

Thirteen of the genes in Cluster A have annotated functions in metal transport and binding, in particular Fe acquisition and homeostasis: *ftn *(ferritin), *so1580 *(TonB-dependent heme receptor), *so1771 *(GntP family permease), *so2045 *(cation efflux family protein), *alcA *(siderophore biosynthesis protein), *so3031 *and *so3032 *(both putative siderophore biosynthesis proteins encoded immediately downstream of *alcA*), *so3033 *(ferric alcaligin siderophore receptor), *so3063 *(sodium:alanine symporter family protein), *so4150 *(putative transporter), *viuA *(ferric vibriobactin receptor), *so4712 *(putative ABC transporter, ATP-binding protein), and *so4743 *(putative TonB-dependent receptor). In contrast to the TonB-dependent receptor genes *so1580 *and *so4743*, either no significant change or a maximal twofold decrease at the 180-min time point was observed for *so2427 *(putative TonB-dependent receptor), located directly upstream of the *so2426 *response regulator gene. Of the 13 genes with transport and binding functions in Cluster A, ten ORFs (*ftn*, *so1580*, *so2045*, *alcA*-*so3031*-*so3032*, *so3033*, *so3063*, *viuA*, *so4743*) were shown in an earlier study to be induced in chromate-challenged wild-type MR-1 cells compared to untreated cells [11,13]. Temporal expression patterns for these genes demonstrated that they were downregulated 2- to 20-fold in the Δ*so2426 *strain over the 180-min time course (Table 1), suggesting that SO2426 acts as a direct or indirect positive regulator of a subset of Fe uptake and storage genes in *S. oneidensis*.

The *alcA*-*so3031*-*so3032 *operon encodes proteins required for siderophore biosynthesis in MR-1 [36], and the product of gene *so3033 *is predicted to allow for the cellular utilization of the structurally undetermined MR-1 siderophore. The differential profiles for these four genes were characterized by a peak in down-regulated expression (ranging from ~7- to 17-fold) at the 180-min time interval post chromate addition, followed by no significant change in expression at 24 h (Table 1). The first gene in the siderophore biosynthetic operon shows 48% sequence identity to *Bordetella pertussis alcA*, which is required for alcaligin production [37], while the two downstream genes (*so3031 *and *so3032*, respectively) diverge from *alcB *and *alcC *and are annotated as putative siderophore biosynthesis genes. While the structural identity of the MR-1 siderophore has not been elucidated, we predict that MR-1 likely produces a siderophore similar to putrebactin [38], a novel cyclic dihydroxamate siderophore characterized from *Shewanella putrefaciens *strain 200 and structurally similar to alcaligin.

**Figure 6:**
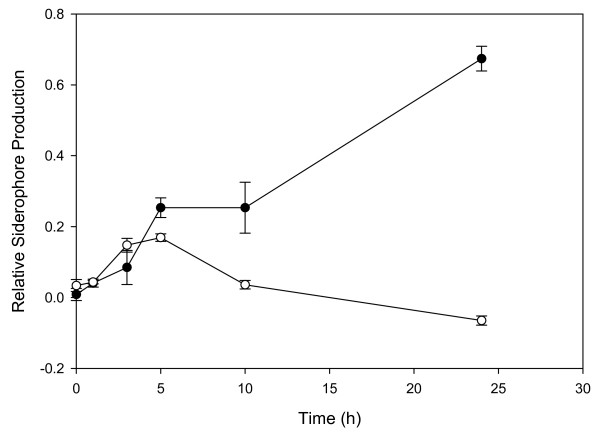
**Relative siderophore production by wild-type MR-1 and Δ*so2426 *mutant cells under chromate conditions**. A final chromate concentration of 0.3 mM was added to mid-log-phase (OD_600_, 0.5) wild-type MR-1 (●) and Δ*so2426 *mutant (○) cultures cultivated in LB broth, and relative siderophore synthesis by each culture was monitored over time using the CAS assay. The relative siderophore production was calculated by subtracting the supernatant *A*_630 _for the wild type or mutant from the uninoculated control and then determining the ratio of corrected supernatant *A*_630 _to control *A*_630_. The graphed values represent mean ratios ± standard errors (bars) for three replicate CAS measurements.

Consistent with the transcriptomic data, further physiological evidence for the involvement of the SO2426 response regulator in controlling siderophore-dependent iron acquisition was obtained by performing semiquantitative liquid CAS assays in which relative siderophore production levels in supernatants of the Δ*so2426 *mutant were compared to those in wild-type MR-1 cultures. Following 24-h growth in LB medium in the absence of added FeCl_3 _or chromate, the Δ*so2426 *mutant (*A*_630_, 0.739 ± 0.02) exhibited essentially no detectable siderophore production over that of the cell-free control (*A*_630_, 0.726 ± 0.005), whereas wild-type MR-1 cultures (*A*_630_, 0.108 ± 0.06) produced approximately 7-fold more siderophore than the Δ*so2426 *mutant. As expected, addition of 50 μM FeCl_3 _to the culture medium reduced siderophore production by the wild-type to near background levels (an approximately 6-fold reduction) but did not affect siderophore accumulation in the Δ*so2426 *mutant. In the presence of 0.3 mM chromate, replicate cultures of wild-type MR-1 exhibited increasing levels in relative siderophore production over time (Figure 6), which was consistent with previous microarray expression data showing induction of siderophore biosynthesis genes in response to chromate exposure [11]. By contrast, the temporal profile for the Δ*so2426 *mutant showed an initial moderate increase and then a dramatic reduction in siderophore excretion compared to the wild type (Figure 6). Collectively, these data support the hypothesis that SO2426 is a positive regulator of siderophore-mediated Fe uptake.

Cluster A also includes an *S. oneidensis *gene that is involved in Fe storage, *i.e*., the *ftn *gene (*so0139*) encoding ferritin. The *ftn *mRNA expression levels were down-regulated 4- to 20-fold in the SO2426 null mutant across the entire temporal range, with the peak in repression occurring at the 180-min time point (Table 1). Although iron is an essential micronutrient for most organisms, free excess Fe in the presence of oxygen is potentially toxic because of its tendency to participate in Fenton chemistry reactions, which generate cell-damaging reactive oxygen intermediates. By scavenging and storing intracellular free Fe in a remobilizable form, Ftn protects against Fe(II)-mediated formation of hydroxyl radicals [39,40]. Studies have indicated that ferritins make important contributions to both Fe storage as well as cellular protection against oxidative stress [41,42] and that *ftn *transcription is co-ordinately regulated by iron and redox stress in some bacteria [43]. *S. oneidensis ftn *was shown previously to be induced at a low level (~2-fold) in chromate-exposed wild-type MR-1 cells in contrast to untreated cells [11]. The induction of *ftn *in *S. oneidensis *might act, at least partially, to mitigate chromate-generated oxidative stress by sequestering excess free Fe available for the catalysis of Fenton reactions and the formation of additional reactive hydroxyl radicals.

### Genes of unknown function

The majority of coordinately repressed genes in Cluster A belong to the functional category of hypothetical and conserved hypothetical proteins (Table 1). Most notably, genes comprising the conserved hypothetical *so1188*-*so1189*-*so1190 *operon exhibited the highest degree of down-regulated expression in the chromate-exposed Δ*so2426 *mutant relative to the wild type, with decreases in mRNA ratios ranging from 45- to > 300-fold over the 180-min period. No significant changes in expression, however, were observed for these genes at the 24-h time point. In addition, the hypothetical gene *so2425*, which is co-transcribed with *so2426 *in a polycistronic transcript (shown in this study), was repressed as much as 11-fold over the 180-min period, pointing to the possibility that the *so2426 *operon may be under positive autoregulatory control. Surprisingly, the functionally unknown genes *so2424*, *so2423*, and *so2422*, which also form part of the *so2426 *operon, showed either a less than 2-fold change or no significant differential expression over the time course.

### Regulatory genes

In addition to metal transport and hypothetical genes described above, the temporal expression profiles for four transcriptional regulatory genes (*so0916*, *so0544*, *cpxR*, *so4567*) were altered in the Δ*so2426 *mutant. The function of the regulators encoded by *so0916*, *so0544*, and *so4567 *in *S. oneidensis *as well as their target genes are not known. However, gene *so4477 *encodes a homolog of CpxR, the response regulator component of the CpxAR envelope stress response system, which has been well characterized in *E. coli *(reviewed in [35,44]). The annotated *cpxR *gene in *S. oneidensis *MR-1 was downregulated as much as threefold in response to the *so2426 *deletion under chromate stress conditions (Table 1). This is in contrast to the two- to three-fold induction measured for *cpxR *in chromate-treated wild-type MR-1 cells [11]. Located immediately downstream of *cpxR *in an apparent operon is *cpxA*, which was downregulated as much as 3-fold in the mutant but grouped in a separate cluster (Cluster B in Figure 5).

In *E. coli*, the CpxAR two-component pair constitutes a stress response system the main function of which is to sense and respond to cell envelope distress, principally misfolded periplasmic proteins, by activating genes encoding proteases and folding catalysts [35,45,46]. However, the complexity, extent, and response overlap of the Cpx system have become progressively apparent, as evident from its involvement in outer membrane porin expression [47], stationary-phase survival [48], and the response to high pH stress [49] to name a few. Although the specific cellular role of the CpxR homolog in *S. oneidensis *has not been established, it is intriguing to consider the possibility that interplay between CpxR and SO2426 may occur in response to metal stress. It is conceivable that the CpxA-CpxR two-component system may respond to oxidative damage imposed on biomolecules as a consequence of reactive oxygen species generated during intracellular partial Cr(VI) reduction.

### Other differentially regulated genes in the *so2426 *deletion mutant

While hypothetical and metal transport/binding genes are dominant in Cluster A (representing 65%), other notable genes with ≥ 2-fold-repressed mRNA levels in the Δ*so2426 *mutant include ORFs codifying a putative bicyclomycin resistance (Bcr) protein (SO2280) and a ferric iron reductase protein (SO3034). Bcr proteins are members of the major facilitator superfamily class of membrane efflux pumps and play important roles in drug resistance [50]. In a previous work, the *so2280 *gene in a wild-type MR-1 genetic background exhibited 2- to 4-fold upregulated expression at the transcript level in response to short-term chromate exposure, *i.e*., up to 90 min [11], but was shown in this study to be repressed as much as 4-fold in Cr(VI)-challenged Δ*so2426 *cells (Table 1), suggesting that the *so2280 *gene is a direct or indirect target of SO2426-mediated activation under chromate conditions. Although the cellular function of SO2280 is presently unknown, its co-ordinately regulated expression with the siderophore biosynthesis operon (*alcA*-*so3031*-*so3032*) and gene *so3033*, encoding a ferric alcaligin siderophore receptor, is intriguing. Studies in other bacterial systems have shown that certain proteins resembling Bcr at the amino acid level function in siderophore export from the cell [51].

Downregulated expression of gene *so3034 *(a putative ferric iron reductase) peaked at the 90-min time point with a 4-fold decrease in mRNA levels (Table 1). Ferric iron reductases are thought to be involved in reducing siderophore-complexed Fe(III) to Fe(II), thus enabling dissociation of the high-affinity Fe^3+^-chelating siderophore from iron [30]. The entire *so3030*-*so3034 *gene cluster, which is predicted to be involved in siderophore production and utilization, exhibits similar temporal expression profiles in the mutant, suggesting a role for the SO2426 response regulator in coordinating these functions. It is not clear whether the other differentially expressed energy metabolism genes (*fdhG*, *fdnH*, *fdhE*, *so4503*, *so4506*, *so4509*) contribute directly to chromate stress response or show altered transcription as a result of secondary effects associated with the *so2426 *deletion. These genes were not observed to be induced in response to chromate exposure in our previous transcriptomic studies using wild-type *S. oneidensis *MR-1 [11].

## Conclusion

*S. oneidensis *SO2426 is an apparent orphan response regulator with an N-terminal domain that contains an aspartate residue at the canonical receiver phosphorylation site and a predicted C-terminal wHTH motif indicative of response regulators in the OmpR subfamily. Although previous work in this laboratory demonstrated enhanced mRNA levels for the *so2426 *gene in a *S. oneidensis *Fur-deficient strain [31] and in chromate-challenged wild-type MR-1 cells [11], the basic molecular function of this predicted transcriptional regulator remained undefined. In this study, by integrating functional genomics and genetic approaches, we have shown that SO2426 is a direct or indirect positive regulator of siderophore-mediated Fe transport systems, the Fe storage gene *ftn*, and other as-yet uncharacterized cation transport genes. At this time, however, we cannot discount the possibility of negative regulation by SO2426 as well. Clustering analysis of temporal transcriptome data revealed a distinctive subset of 46 coordinately expressed genes that were consistently downregulated in the *so2426 *deletion mutant during the initial 180-min period of chromate exposure. Many of these genes – namely, *ftn*, the *alcA*-*so3031*-*so3032 *siderophore biosynthesis operon, *so1580 *(TonB-dependent heme receptor), *so4743 *(TonB-dependent receptor), *viuA*, *so2280 *(*bcr*), as well as a number of hypothetical genes – constituted the predominant molecular response to acute chromate stress in wild-type MR-1 [11]. The transcriptomic analyses described here identified a number of possible directly SO2426-regulated genes, including those of unknown function, that may serve as targets for future studies.

One possible hypothesis that can be generated from the collective data described here is that *S. oneidensis *MR-1 employs at least two control mechanisms for regulating Fe acquisition via siderophores under conditions of external Fe insufficiency: derepression as a result of decreased Fur activity and activation by SO2426 to enhance needed expression of Fe(III) transport systems. CAS siderophore assays clearly demonstrated that the Δ*so2426 *mutant was deficient in siderophore production compared to wild-type MR-1 cultures in the presence and absence of chromate, indicating that SO2426 plays a positive role in coordinating the regulation of siderophore-dependent Fe uptake mechanisms in *S. oneidensis*. Furthermore, the impaired chromate reduction rate of the *so2426 *deletion strain points to a connection between Fe transport and chromate stress response that requires further delineation at the molecular level. Our previous global studies characterizing the transcriptome and proteome of wild-type MR-1 in response to sub-lethal acute doses of chromate demonstrated a dramatic and unexpected induction of genes involved in Fe sequestration and uptake in contrast to unchallenged cells [11,13]. Two possible explanations for this observation are (i) Fe limitation under external chromate conditions, and (ii) direct or indirect displacement of the Fe(II) cofactor from Fur-binding sites under elevated Cr(VI), resulting in decreased DNA-binding activity of the Fur repressor and subsequent derepression of target genes. Exposure to other metals has been shown to perturb intracellular Fe pools and hence influence Fur-mediated repression [52]. Future studies will focus on experimentally confirming direct gene targets of SO2426 control and further elucidating the linkage between regulation of Fe homeostasis and chromate stress response at molecular and physiological levels.

## Methods

### Bacterial strains, plasmids, and growth conditions

All bacterial strains and plasmids used in this study are listed in Table 2. *S. oneidensis *strain MR-1 [4,53] was used as the wild type. The Δ*so2426 *strain, a derivative of MR-1, contains an in-frame deletion of the *so2426 *locus on the chromosome (see below for a detailed description of mutant construction). For growth studies, wild-type and mutant strains were cultivated aerobically with shaking (250 rpm) at 30°C in Luria-Bertani (LB) medium (pH 7.2) alone or in media amended with 0.3 mM (final concentration) of K_2_CrO_4_. Optical density was monitored at a wavelength of 600 nm (OD_600_) in triplicate using either a Spectronic 20D+ spectrophotometer (Thermo Electron Cooperation, Waltham, MA) or the Bioscreen C microbiological culture system (Growth Curves USA, Piscataway, NJ) as described elsewhere [36].

**Table 2:** Bacterial strains and plasmids used in this study

**Strain or plasmid**	**Relevant characteristics**	**Source or reference**
**Strains**		

*S. oneidensis*		
MR-1 (ATCC 700550)	Wild type	Laboratory Stock
MR-1/Δ*so2426*	In-frame deletion of the *so2426 *locus	This study
MR-1/Δ*so2426*(*so2426*^+^)	Δ*so2426 *mutant complemented with pBBRso2426	This study
*E. coli *WM3064	DAP-auxotrophic wild type	[32]

**Plasmids**		

pJK100	Allelic-exchange vector	[32]
pJK102	pJK100 with *so2426 *upstream and downstream regions	This study
pCM157	Cre recombinase expression vector	[32]
pBBR1MCS-5	Gm^r^, broad-host range vector, *lacPOZ'*	[56]
pBBRso2426	pBBR1MCS5-based construct harboring entire *so2426 *gene	This study

For transcriptome analyses, three separate batch cultures each of wild-type MR-1 and the Δ*so2426 *mutant were grown to mid-exponential phase (OD_600_, 0.5) in 100 ml of LB broth, followed by addition of 2 M K_2_CrO_4 _to a final concentration of 0.3 mM. The two different sets of chromate-challenged cultures (wild type and mutant), each consisting of three biological replicates, were grown aerobically in parallel at 30°C. Cells were harvested for RNA extraction at 5, 30, 60, 90, 180 min, and 24 h post-chromate exposure. For RT-PCR and 5' RACE experiments, wild-type *S. oneidensis *MR-1 cells were grown to mid-log phase (OD_600_, 0.5) in LB broth at 30°C and then exposed to chromate (final concentration, 1 mM) for 30 min before harvesting cells for RNA extraction. Untreated MR-1 cells were grown in parallel and used as the control.

### Chromate reduction and CAS siderophore assays

Disappearance of extracellular Cr(VI) was quantified spectrophotometrically using the 1,5-diphenylcarbazide (DPC) method as described elsewhere [34]. Cultures were assayed at different time points (5, 30, 60, 90, 180 min, and 24 h) post chromate-challenge to determine the amount of residual Cr(VI) remaining in the medium by measuring absorbance at 540 nm using a Varian (Cary-1E) UV-visible spectrophotometer (Hewlett-Packard, Wilmington, DE). Cell-free LB medium containing 0.3 mM K_2_CrO_4 _served as the abiotic negative control and was monitored in parallel with the experimental samples.

The chrome azurol S (CAS) assay for detection of siderophore production was performed essentially as described by Schwyn and Neilands [54]. Siderophore biosynthesis and excretion by the Δ*so2426 *mutant was compared with those by the wild-type MR-1 strain in LB broth with or without the addition of 50 μM FeCl_3 _or 0.3 mM K_2_CrO_4_. Cultures of these strains were grown aerobically to stationary phase (OD_600 _> 1.0) at 30°C for 24 h. Cell-free supernatants of *S. oneidensis *cultures were mixed 1:1 with the CAS assay solution and equilibrated at room temperature for 2 h before the absorbance at 630 nm was measured. The relative siderophore production was calculated as the ratio of the control (uninoculated LB medium) *A*_630 _to the wild-type or mutant strain supernatant *A*_630_. All CAS measurements were performed in triplicate, and at least two independent determinations were conducted.

### Construction of an *S. oneidensis so2426 *deletion strain and complementation

An *S. oneidensis *MR-1 in-frame deletion mutant lacking the *so2426 *locus was generated using the *cre-lox *recombination system as described elsewhere [32,33]. The application of this mutagenesis strategy to targeted *S. oneidensis *MR-1 genes has been described previously [55]. The primer sequences used in the generation and verification of the Δ*so2426 *mutant are listed in Table 3. PCR was used to amplify a 900-bp region upstream and a 840-bp region downstream of the *so2426 *gene using primer pairs Del900-F/Del900-R and Del840-F/Del840-R, respectively. The amplified regions were cloned into the kanamycin-resistant (Km^r^) plasmid pJK100 [32]. The resulting plasmid, pJK102, carrying a correct construct was introduced into the *E. coli *strain WM3064 [32], a diaminopimelic acid (DAP) auxotroph, and then subsequently moved into MR-1 via conjugation with WM3064. The resultant MR-1 strain was Km^r^/Tet^s ^and contained the *loxP-Km*^*r*^-*loxP *cassette. Removal of the kanamycin cassette and the helper plasmid was performed as described elsewhere [33]. A cre-recombinase enzyme-producing plasmid, pCM157/Tet^r^, was introduced into MR-1 to resolve the *loxP-Km*^*r*^-*loxP *cassette through conjugation. The Tet^r ^plasmid pCM157 was cured from MR-1 by continuous culturing in non-selective LB medium. The in-frame deletion of *so2426 *was confirmed by PCR amplification using several sets of primers (ISU2426/ISD2426, OP840, OP900, kanF/kanR in Table 3) and DNA sequencing.

**Table 3:** Oligonucleotide primers used in this study

**Primer**	**DNA sequence (5' → 3')**	**Reference or source**
**Construction of Δ*so2426 *strain**		

Del840-F	CTTGGTTACCGGCTAGTGAAC	This study
Del840-R	GGCAGGTATTGATAACAATGA	This study
Del900-F	GGTTCACACCAATCGCATTAG	This study
Del900-R	TGGCCAATACCCGCTTACCGC	This study
ISU2426	GCCTAAGATGCCATCAGT	This study
ISD2426	TCTTCAAGATTTAGCTTATCC	This study
OP840	CACATAAGGCAGACCTTCGTC	This study
OP900	ATGGTCCGTACTGTGGCCGC	This study
kanF	ATTGTTGATGCGCTGGCAGT	[32]
kanR	TCCGGTGAGAATGGCAAAAG	[32]

**Construction of complementation plasmid**		

2426com-F	ACACACAAGCTTGCGCTTTTCTTTTAGGTACAA	This study
2426com-R	ACACACGGATCCGACTCACAGAGGGCGCTTA	This study

**RT-PCR analysis**		

P1	ATGATATTGATTCTCGTTTG	This study
P2	CCGAGTGTGGTTAACTGATG	This study
P3	CGCCGAGTATTACTGATATGC	This study
P4	AAGCGCTAAAACTGTATCC	This study
P5	TTAACATGCATCTACTTTTA	This study
P6	GATCTTGCAGGTTGTTGTT	This study
P7	TCATACACTCTTTCGCTTAT	This study
P8	CTGTTTCTTCAACTCAGCCT	This study
P9	CAGTCGTTAGCTCAATTGCT	This study

The Δ*so2426 *mutant was complemented by reintroduction of the wild-type *so2426 *allele on a low-copy-number plasmid. For this, the *so2426 *open reading frame with 100 bp of upstream sequence was amplified from *S. oneidensis *MR-1 genomic DNA using primers 2426com-F and 2426com-R (Table 3). The PCR product was gel purified, digested, and ligated into the *Bam*H1 and *Hind*III sites of the plasmid pBBR1MCS-5 [56], which contains a gentamicin resistance (Gm^r^) cassette. The resultant construct harboring the complete *S. oneidensis so2426 *gene with its endogenous promoter was designated pBB2426. The pBB2426 plasmid was electroporated into *E. coli *strain WM3064 and then introduced into the *S. oneidensis *Δ*so2426 *mutant and wild-type MR-1 strains via conjugation. In addition, plasmid pBBR1MCS5 without the insert (empty vector) was also transferred into the mutant and wild-type strains via conjugation with WM3064. Gm^r ^*Shewanella *colonies were verified for the presence of the *so2426 *gene by PCR analysis and DNA sequencing.

### RNA isolation

For microarray profiling and real-time RT-PCR experiments, total cellular RNA was isolated from *S. oneidensis *cultures using the TRIzol reagent (Invitrogen, Carlsbad, CA) according to the manufacturer's instructions. RNA preparations were treated with RNase-free DNase I (Ambion Applied Biosystems, Foster City, CA) to remove residual genomic DNA and subsequently further purified using the RNeasy Mini Kit (Qiagen, Valencia, CA) according to the manufacturer's RNA cleanup protocol. Total RNA was quantitated as described previously [36]. For RT-PCR and 5'-RACE analyses, total RNA was extracted from *S. oneidensis *cultures using the RNeasy Mini Kit (Qiagen). Chromosomal DNA contamination was removed by incubating total purified RNA with 3 U of RNase-free DNase (Ambion Applied Biosystems) for 30 min at 37°C. The DNA digestion reaction was performed twice for each RNA sample. The quantity and purity of the RNA was assessed as described previously [36]. The integrity of all RNA samples was assessed visually using 1% agarose gel electrophoresis and ethidium bromide staining.

### Microarray hybridizations and data analysis

Transcriptome analyses were performed with a microarray containing 4,197 PCR amplicons and 451 specific 50-mer oligonucleotides, covering approximately 94% of the total predicted gene content of *S. oneidensis *MR-1. Fabrication of the MR-1 arrays has been described in detail elsewhere [57]. Synthesis of the two differentially labeled cDNA pools (wild-type MR-1 and the Δ*so2426 *mutant) to be compared, microarray prehybridization and hybridization, and post-hybridization washings were performed as described previously [36]. Temporal gene expression analysis was performed using six independent microarray hybridizations (three biological replicates × two dye-swap reactions) for each of six time points, with each slide containing two spots representing each gene at different array locations for a total of 12 signal intensity measurements per gene per time point. Image quantification, data normalization, and analysis of gene expression data for statistical significance were conducted as described by Brown *et al*. [36]. The time-series microarray expression profiles of the Δ*so2426 *mutant relative to the parental strain were clustered using Hierarchical Clustering Explorer (HCE) [58]. During the clustering process, only genes with an expression value of at least ≥ 2-fold or ≤ 0.5-fold in one or more of 6 expression profiling time points were included in the analyses. As a result, a dataset of 841 genes was clustered based on average linkage using Euclidean distance.

### Access to microarray data

The microarray data reported in this study have been deposited in MIAME-compliant format at Gene Expression Omnibus on the NCBI website [59] under series accession number GSE12129. The statistically analyzed microarray output is provided in Additional file 1.

### Quantitative real-time RT-PCR

Reverse transcriptase, quantitative real-time PCR (qRT-PCR) was used to provide an independent assessment of gene expression for five selected genes (*so0404*, *so2426*, *so3670*, *so1826*, and *so3585*), which exhibited different expression patterns (*i.e*., down-regulated or no change). These genes are predicted to encode a hypothetical protein (SO0404), a DNA-binding response regulator (SO2426), proteins involved in transport and binding of cations (SO3670 and SO1826), and a putative azoreductase (SO3585). Relative expression patterns for each selected gene were independently confirmed using the following primer pairs, which were designed using the program Primer3 : SO0404, 5'-AGTATAACCAAGCGCCAGTA and 3'-GCATCGGTATTAACTTGCTC; SO2426, 5'-GCAGAAGGATTTAGGTCGAT and 3'-CGGTGTTGATTAAAGTACGC; SO3670, 5'-TCTAAACAGTCGCAGGAGCA and 3'-GCGCCATATTGCTATCCATT; SO1826, 5'-GGGTGTCCCAAGCTAGT CAA and 3'-GAGCATTACTCGTCCCCTGA; SO3585, 5'-CGAGGCTATCCATC ACTTAG and 3'-TGGAAAACACGATAAAGACC. Reverse transcription was performed on the 180-min time point samples with 3 μg of total cellular RNA and 2.5 mM random hexamers using Superscript™ II RNase H^- ^Reverse Transcriptase (Invitrogen) as described previously [31]. Quantitative PCR was carried out in an iCycler iQ^® ^real-time PCR system (BioRad, Hercules, CA) in 50-μl reaction mixtures containing 1 μl cDNA, 600 nM forward and reverse primers, and iQ SYBR green supermix (BioRad) according to the manufacturer's instructions and conducted under the following conditions: 30 sec at 95°C, followed by 40 cycles of 15 sec at 95°C, 30 sec at the specific annealing temperature, and 30 sec at 72°C. The qRT-PCR reactions were performed in triplicate for each of the three biological replicates tested. Standards for each gene of interest were included in the analysis as described previously [31]. The final Δ*so2426*/WT ratio for each target gene and the standard error were calculated as described elsewhere [31], and the linear correlation between the qRT-PCR and microarray data was determined based on the log mean values using Sigma-Plot version 9.0 (SPSS Inc., Chicago, IL).

### RT-PCR and 5'-RACE analyses

Reverse transcription-PCR (RT-PCR) was performed to investigate the transcription of the *so2427*-*so2422 *gene cluster on the MR-1 chromosome. First-strand cDNA was synthesized from 1 μg of DNase-treated total RNA isolated from MR-1 cultures grown in LB broth under either non-stress (no chromate added) or chromate stress (1 mM, 30-min exposure) conditions. Random hexamers (250 ng), dNTPs (10 mM), 5× First-Strand buffer (4 μl), 0.1 M DTT (1 μl), RNaseOUT (1 μl), recombinant RNase inhibitor (40 U), and Superscript™ II Reverse Transcriptase (200 U; Invitrogen) were added to the reaction mixture, which was incubated at 25°C for 10 min, 42°C for 50 min, and then 70°C for 15 min in accordance with the recommendations of the supplier. Synthesized cDNA was used as the template in subsequent PCR amplification reactions with gene-specific oligonucleotide primers P1–P9 (Table 3) spanning the *so2427*-*so2422 *gene region. The TripleMaster PCR System (Eppendorf, Westbury, NY) was used for amplification, with primers added to each reaction mixture at a final concentration of 250 nmol. The reaction mixtures were subjected to 30 cycles of denaturation at 94°C for 60 s, annealing at 44–52°C (depending on the primer T_m_) for 60 s, and an extension at 72°C for 1–2 min in an Eppendorf Mastercycler ep gradient thermocycler. Each amplified product was analyzed by 1% agarose gel electrophoresis with ethidium bromide staining. In addition, each PCR product obtained was cloned into the pGEM-T vector (Promega, Madison, WI) and sequenced at Purdue University's Low-Throughput DNA Sequencing Laboratory to confirm that they were the expected loci of each respective gene region.

The transcriptional start site of the *so2426 *gene was localized using the 5' RACE System for Rapid Amplification of cDNA Ends version 2.0 (Invitrogen) according to the manufacturer's instructions. Briefly, cDNA was generated using Superscript™ II reverse transcriptase (42°C, 1 h; Invitrogen) in a reaction containing 2 μg of total cellular RNA (from non-stressed or chromate-stressed MR-1 cells) and a *so2426*-specific primer (2426-GSP1: 5'-TAACCGATTGAATTGTT-3'). Nested PCR was performed using two *so2426*-specific primers (2426-GSP2: 5'-TGCACTAACCGTCGCTCTATGGCCTGCAAA-3' and 2426-GSP3: 5'-GCACCAAGTTCATAGCTTCGTATCCTGTCT-3') and the manufacturer-supplied abridged anchor and abridged universal anchor primers. The 5'-RACE products were analyzed by agarose gel electrophoresis and cloned into the pGEM-T vector (Promega) prior to DNA sequencing.

## Authors' contributions

KC created the deletion mutant analyzed in this work; performed the microarray experiments, qRT-PCR verifications, and the initial data analysis; and carried out the growth studies and Cr(VI) reduction assays. WW performed the operon structure analysis, transcription start site determination, and CAS siderophore assays. X–FW carried out statistical and clustering analyses of the microarray data. KC and X–FW both contributed to the manuscript writing. DKT conceived and coordinated the study and performed the majority of the manuscript writing. All authors read and approved the final manuscript.

## Supplementary Material

Additional file 1 **Complete processed microarray dataset for the *S. oneidensis *Δ*so2426 *mutant compared to the wild type under chromate conditions**. This file provides the complete statistically analyzed microarray output for all six time points (5, 30, 60, 90, 180 min, and 24 h) during chromate (0.3 mM) exposure starting with the Gene ID, mean Δ*so2426 *mutant/WT expression ratio, *n *(number of signal values out of 12 total per gene included in the statistical analysis), statistical significance, gene name, gene product, main role category, and subrole category. ArrayStat™ (Imaging Research, Inc., Ontario, Canada) was used to determine the common error of normalized expression values, remove outliers, and determine statistical significance via a *z *test for two independent conditions and the false discovery rate method (nominal α, confidence cutoff of *p *< 0.05). Click here for file
